# Supplementary material for: Pyrrolysine Aminoacyl-tRNA Synthetase as a Tool for Expanding the Genetic Code
Source: Int J Mol Sci. 2025 Jan 10;26(2):539. doi: 10.3390/ijms26020539 (PMC11764691; doi:10.3390/ijms26020539)
Supplement: Supplementary file 1 [file ijms-26-00539-s001.zip › ijms-3367630-supplementary.pdf]

Enzymes	Amino acids	K_m, µM × 10^3	k_{cat}, s^{-1} × 10^{-2}	k_{cat}/K_m, $µM^{-1}·s^{-1}$ × 10^{-5}	Relative catalytic efficiency	UAG translation efficiency, %	Source of information
MmPylRS	Pyl	0.02 ± 0.004	0.83 ± 0.03	41.5	100	15± 1.5	L. Guo, Y. Wang, A. Nakamura, D. Eiler, J.M. Kavran, M. Wong, L.L. Kiessling, T.A. Steitz, P. O'Donoghue, D. Söll, Polyspecific pyrrolysyl-tRNA synthetases from directed evolution, Proc. Natl. Acad. Sci. U.S.A. 111 (47) 16724-16729, https://doi.org/10.1073/pnas.1419737111 (2014).
MbPylRS	Pyl	0.02 ± 0.002	3.01 ± 0.04	151	364	—	
IFRS	3-I-Phe	0.44 ± 0.04	0.44 ± 0.02	1.00	2.4	48± 1.2	
	3-Br-Phe	0.62 ± 0.24	0.48 ± 0.03	0.77	1.9	68 ± 5.2	
	3-Cl-Phe	1.36 ± 0.13	0.76 ± 0.05	0.56	1.3	46 ± 3.2	
	3-CF_3-Phe	0.45 ± 0.09	0.28 ± 0.01	0.62	1.5	53 ± 2.3	
	3-Me-Phe	0.95 ± 0.17	0.34 ± 0.05	0.36	0.9	30 ± 0.5	
	3-MeO-Phe	1.64 ± 0.19	0.07 ± 0.01	0.04	0.1	<1	
	3-Br-ThA	0.37 ± 0.09	0.14 ± 0.01	0.38	0.9	7 ± 0.9	
PylRS	BocK	0.46 ± 0.084	1.10 ± 0.043	2.39	100	—	Jiang HK, Weng JH, Wang YH, Tsou JC, Chen PJ, Ko AA, Söll D, Tsai MD, Wang YS. Rational design of the genetic code expansion toolkit for in vivo encoding of D-amino acids. Front Genet. 2023 Oct 13;14:1277489. doi: 10.3389/fgene.2023.1277489
DFRS2	D-CF_3-Phe	1.65 ± 0.210	0.07 ± 0.001	0.04	1.7	—	
	L-CF_3-Phe	0.09 ± 0.012	0.49 ± 0.035	5.44	228		
	D-Cl-Phe	1.20 ± 0.112	0.11 ± 0.005	0.09	3.8		
	L-Cl-Phe	0.05 ± 0.004	0.23 ± 0.005	4.60	192		
	D-Br-Phe	0.81 ± 0.164	0.10 ± 0.006	0.12	5.0		
	L-Br-Phe	0.12 ± 0.010	0.33 ± 0.015	2.75	115		

Enzymes	k_{cat}, $s^{-1} \times 10^{-3}$	K_M ^{ATP}, µM	K_M ^{tRNA}, µM	K_M ^{BocK}, mM	k_{cat}/K_M ^{tRNA}, $\mu M^{-1} \cdot s^{-1} \times 10^{-3}$	Relative catalytic efficiency	Source of information
chPylRS	11.88 ± 0.18	2.54 ± 0.16	0.26 ± 0.07	1.03 ± 0.05	45.69	1	Bryson DI, Fan C, Guo LT, Miller C, Söll D, Liu DR. Continuous directed evolution of aminoacyl-tRNA synthetases. Nat Chem Biol. 2017 Dec;13(12):1253-1260. doi: 10.1038/nchembio.2474. Epub 2017 Oct 16. Erratum in: Nat Chem Biol. 2018 Jan 16;14(2):186. doi: 10.1038/nchembio0218-186.
V31I, T56P	73.15 ± 1.01	5.74 ± 0.20	0.10 ± 0.02	0.82 ± 0.18	731.50	15.9	
V31I, T56P, A100E	110.23 ± 4.65	3.45 ± 1.19	0.13 ± 0.03	0.91 ± 0.08	847.92	18.4	
V31I, T56P, H62Y, A100E	103.87 ± 2.37	3.96 ± 0.52	0.05 ± 0.01	1.13 ± 0.23	2,077.40	45.2	

Table 1: Kinetic parameters of native and mutated PylRS variants for aminoacylation.

Enzymes	Amino acids	K_m, $\mu M \times 10^3$	k_{cat}, $s^{-1} \times 10^{-2}$	k_{cat}/K_m, $\mu M^{-1} \cdot s^{-1} \times 10^{-5}$	Relative catalytic efficiency	UAG translation efficiency	Source of information
MmPylRS	Pyl	0.05 ± 0.008	29.8 ± 1.2	596	100	15± 1.5	L. Guo, Y. Wang, A. Nakamura, D. Eiler, J.M. Kavran, M. Wong, L.L. Kiessling, T.A. Steitz, P. O'Donoghue, D. Söll, Polyspecific pyrrolysyl-tRNA
MbPylRS	Pyl	0.055 ± 0.005	10.5 ± 1.1	191	32	—	
AcKRS1	3-I-Phe	6.14 ± 0.32	12.6 ± 1.5	2.05	0.34	19 ± 2.0	
	3-Br-Phe	11.9 ± 0.30	13.6 ± 0.35	1.14	0.19	3 ± 0.5	
	3-Cl-Phe	9.46 ± 3.85	8.54 ± 2.73	0.90	0.15	<1	

	3-CF_3-Phe	1.09 ± 0.28	3.91 ± 0.48	3.59	0.60	3 ± 0.3	synthetases from directed evolution, Proc. Natl. Acad. Sci. U.S.A. 111 (47) 16724-16729, https://doi.org/10.1073/pnas.1419737111 (2014).
	3-Me-Phe	0.80 ± 0.19	1.37 ± 0.19	1.71	0.29	<1	
	3-MeO-Phe	6.27 ± 1.17	4.15 ± 0.35	0.66	0.11	<1	
	CF_3-AcK	0.61 ± 0.06	1.24 ± 0.02	2.03	0.34	3 ± 0.2	
	AcK	35.3 ± 10.9	3.23 ± 0.61	0.09	0.02	2 ± 0.1	
	Pyl	nd	nd	—	—	—	
AcKRS3	AcK	137 ± 59	14.6 ± 3.8	0.11	0.02	4 ± 0.2	
	3-I-Phe	16.6 ± 1.9	34.3 ± 2.4	2.07	0.35	21 ± 2.1	
	3-Br-Phe	22.4 ± 2.0	26.3 ± 1.0	1.17	0.20	5 ± 0.1	
	3-CF_3-Phe	3.21 ± 0.97	4.68 ± 0.39	1.46	24	6 ± 0.3	
IFRS	3-I-Phe	0.82 ± 0.09	8.71 ± 0.33	10.6	1.8	48 ± 0.1	
	3-CF_3-Phe	1.13 ± 0.10	7.29 ± 0.21	6.45	1.1	53 ± 2.3	
	3-Br-ThA	1.57 ± 0.32	4.43 ± 0.27	2.82	0.47	7 ± 0.9	
PylRSc	BocK	0.84 ± 0.06	12.93 ± 0.62	15.39	100	—	Jiang HK, Weng JH, Wang YH, Tsou JC, Chen PJ, Ko AA, Söll D, Tsai MD, Wang YS. Rational design of the genetic code expansion toolkit for in vivo encoding of D-amino acids. Front Genet. 2023 Oct 13;14:1277489. doi: 10.3389/fgene.2023.1277489.
DFRSc	D-CF_3-Phe	ND	ND	—	—		
	L-CF_3-Phe	7.12 ± 1.67	10.43 ± 0.37	1.46	9.5		
	D-Cl-Phe	ND	ND	—	—		
	L-Cl-Phe	5.38 ± 1.28	11.18 ± 0.29	2.08	13.5		
	D-Br-Phe	ND	ND	—	—		
	L-Br-Phe	9.80 ± 1.65	10.21 ± 0.35	1.04	6.8		

Table 2: Kinetic parameters of native and mutated PylRS variants for amino acid activation.
